# Estimating Gaits of an Ancient Crocodile-Line Archosaur Through Trajectory Optimization, With Comparison to Fossil Trackways

**DOI:** 10.3389/fbioe.2021.800311

**Published:** 2022-02-03

**Authors:** Delyle T. Polet, John R. Hutchinson

**Affiliations:** Structure and Motion Lab, Comparative Biomedical Sciences, Royal Veterinary College, Hatfield, United Kingdom

**Keywords:** locomotion, predictive simulation, Pseudosuchia, fossil trackways, energetics, Chirotheriidae

## Abstract

Fossil trackways provide a glimpse into the behavior of extinct animals. However, while providing information of the trackmaker size, stride, and even speed, the actual gait of the organism can be ambiguous. This is especially true of quadrupedal animals, where disparate gaits can have similar trackway patterns. Here, predictive simulation using trajectory optimization can help distinguish gaits used by trackmakers. First, we demonstrated that a planar, five-link quadrupedal biomechanical model can generate the qualitative trackway patterns made by domestic dogs, although a systematic error emerges in the track phase (relative distance between ipsilateral pes and manus prints). Next, we used trackway dimensions as inputs to a model of *Batrachotomus kupferzellensis*, a long-limbed, crocodile-line archosaur (clade Pseudosuchia) from the Middle Triassic of Germany. We found energetically optimal gaits and compared their predicted track phases to those of fossil trackways of *Isochirotherium* and *Brachychirotherium*. The optimal results agree with trackways at slow speeds but differ at faster speeds. However, all simulations point to a gait transition around a non-dimensional speed of 0.4 and another at 1.0. The trackways likewise exhibit stark differences in the track phase at these speeds. In all cases, including when simulations are constrained to the fossil track phase, the optimal simulations after the first gait transition do not correspond to a trot, as often used by living crocodiles. Instead, they are a diagonal sequence gait similar to the slow tölt of Icelandic horses. This is the first evidence that extinct pseudosuchians may have exhibited different gaits than their modern relatives and of a gait transition in an extinct pseudosuchian. The results of this analysis highlight areas where the models can be improved to generate more reliable predictions for fossil data while also showcasing how simple models can generate insights about the behavior of extinct animals.

## Introduction

Despite the incredible animal diversity of the present, the past contains forms with no ideal modern analogues. *Batrachotomus kupferzellensis* (Gower, 1999) is one such example. This long-limbed, crocodile-line archosaur (clade Pseudosuchia) from the Middle Triassic of Germany had a large head and a massive tail similar to modern crocodylians but a more erect (adducted) limb posture similar to modern (cursorial) mammals (Gower and Schoch, 2009). Was *Batrachotomus* more similar to a mammal in its locomotion, to a modern crocodylian, or was it altogether different?

Bonaparte (1984) considered the erect limbs, and elongated pubis and ischium, of “rauisuchids” (a group containing *Batrachotomus*) to be adaptations for parasagittal locomotion that enabled them to survive a Middle–Late Triassic faunal replacement. Parrish (1986) postulated that the erect limbs of “rauisuchians” and other archosaurs gave them increased maneuverability on land as in mammals. While Parrish noted that their hindlimb plantigrady and crurotarsal ankle were more similar to those of modern crocodylians, reorganization of the ankle resulted in symmetrical pull of the plantarflexors, leading to simple plantarflexion of the ankle rather than lateral rotation with plantarflexion as seen in crocodylians and lizards. Nesbitt et al. (2013) interpreted plantigrady and the extended calcaneal tuber as adaptation to high power rather than high speed. Apart from a consensus that *Batrachotomus* used parasagittal and erect locomotion, and was quadrupedal (Bishop et al., 2020), there is as yet no analysis on the kind(s) of gait it may have employed.

Many aspects of gait choice in cursorial, quadrupedal mammals emerge from work-based optimization in simple parasagittal models. Trajectory optimization, in particular, allows for the numerical optimization of continuous motion through time. Xi et al. (2016) used trajectory optimization to recover the four-beat walk and trot typically used by mammals with a planar model. Galloping was later discovered as optimal when a compliant torso was added (Yesilevskiy et al., 2018). By minimizing mechanical work in parasagittal models, the body’s pitch moment of inertia (when normalized to glenoacetabular distance and mass as the Murphy number), was shown to broadly determine which mammals do and do not trot (Usherwood, 2020; Polet, 2021b).

Precise anatomical details are often unnecessary for the biomechanical models to arrive at similar solutions to the animals they are based upon. For example, a model with one rigid body element, massless prismatic legs, and no elastic elements captured walking and trotting in dogs, matching the gait transition speed, changes in the duty factor with speed, ground reaction force shape, and limb phase with reasonable accuracy from minimizing a cost combining limb work with a penalty for rapid changes in force (Polet and Bertram, 2019). Such models may be useful in paleontological research, where soft-body details including musculature geometry, fiber length, and tendon length are not usually preserved. Indeed, the gaits of fossil organisms are often very difficult to be determined from trackways, although some attempts have been broadly successful (see Nyakatura et al., 2019; Vincelette, 2021).

**FIGURE 1:**
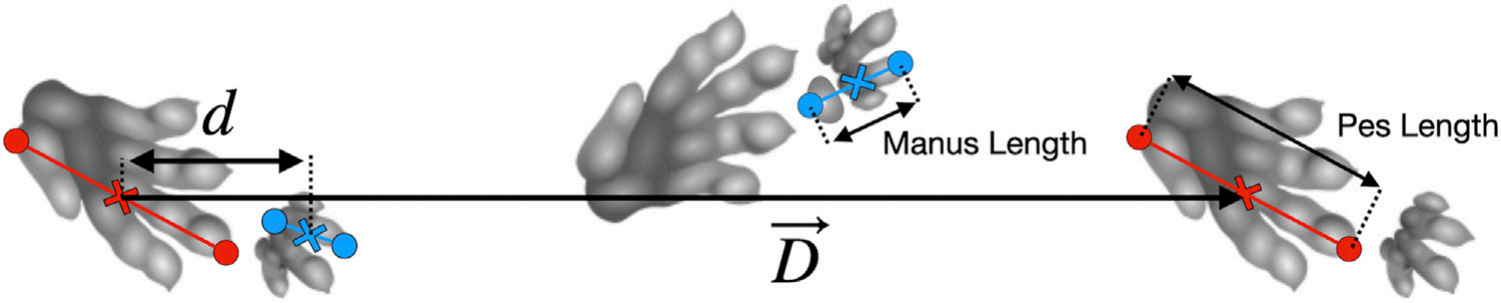
Schematic of a *Brachychirotherium* trackway, showing definition of parameters. Footprints are measured from the caudal-most point to the tip of digit III, or to digit II in the case of *Brachychirotherium* manus. The caudal side of digit V was used (e.g., the second manus in the diagram) when available. The manus–pes distance is calculated between midpoints along the stride vector, $\vec{D}$ ; the track phase is then d/D . Trackway adapted from Wikimedia Commons (2010), used here under a CC BY-SA 3.0 license.

Triassic pseudosuchian fossil trackways (referred to ichnotaxa *Isochirotherium* and *Brachychirotherium*) may belong to *Batrachotomus* or a close relative (Petti et al., 2009; Diedrich, 2015; Apesteguía et al., 2021; Klein and Lucas, 2021), and offer information on an approximate trackmaker size and gait parameters (e.g., track phase, defined as ipsilateral pes to manus displacement divided by the stride length, Figure 1). However, the precise gait employed by these trackmakers remains ambiguous.

A biomechanical model can provide quantitative predictions of the track phase given the stride length and trackmaker size. If the model adequately predicts the track phase, it offers not only evidence that a particular gait was used but also clues about other aspects of the trackmaker’s locomotion not directly recorded in fossil trackways.

## Methods

### Planar Model and Optimization Scheme

**FIGURE 2:**
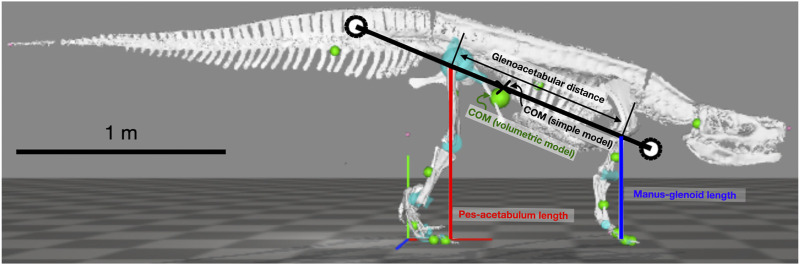
Simple quadrupedal model used for simulations overlayed on the skeletal model used for length measurements. White circles show radius of gyration from the center of mass (x). Only two of four legs (red and blue lines) are shown in the figure.

The parasagittal, planar model follows the study by Polet and Bertram (2019); Polet (2021b). It has a rigid trunk and massless legs that push along an axis connecting footfall location to acetabulum or glenoid through time (Figure 2). Feet are simple points, actuator force is instantaneously reflected in ground reaction force, and sliding friction is infinite. The center of mass (COM) lies along the glenoacetabular axis, and the pitch moment of inertia (MOI), stride length ( D ), and average horizontal speed are provided to the model.

The optimizations minimize an objective J  combining the axial limb work, with a regularization penalizing rapid changes in force, as follows:

$$\begin{array}{c} J=\underset{i}{\sum }\overset{T}{\underset{0}{\int }}F_{i}\dot{L}+c\dot{F_{i}^{2}}dt \end{array},$$ (1)

where $F_{i}$ is the axial force of the *i*th limb, L is the axial limb length, c is the force-rate penalty parameter, and 0 ≤t ≤T is time during a stride. The penalty on the force rate ensures no discontinuity in force or velocity (i.e., no collisions). However, as force rates are decision variables, near-impulsive solutions (i.e., quasi-collisions with sharp peaks in force) are possible and emerge when force-rate penalties are low.

In the optimization scheme, c is normalized as $c\prime =c\frac{Mg}{L_{B}T}$ , where $L_{B}$ is the glenoacetabular distance, M is the body mass, and g is the acceleration due to gravity (Polet and Bertram, 2019). Note that throughout this paper, we use the prime symbol to denote dimensionless variables. Values of c′ are reported here for $U_{H}^{\prime }=0.4$ ( $U_{H}^{\prime }=U/\sqrt{gH}$ , where U is the average horizontal speed, g is the acceleration due to gravity, and H is the hip height) and were scaled appropriately according to T (corresponding to the same c ).

Force rates are controls in the model, with forces, velocities, and positions serving as states. A leg-impulse state is added to prevent simultaneous cranial and caudal contact of a single leg. Footfall positions are parameters (and decision variables) for the optimization. Additional relaxation parameters and slack variables are added as controls to regularize complementarity conditions (see Polet and Bertram, 2019 for details).

As the gaits of interest are symmetrical, the simulation is over a half cycle (time from 0 to *T/2*). An initial and final COM horizontal position of 0 and *D/2* is imposed. A periodicity constraint is enforced such that the body kinematics must be equal at t=0 and T/2 , and left-side forces at t=0 must be equal to right-side forces at t=T/2 . The vertical position of hips and shoulders are constrained to be above ground, and the orientation of the torso (relative to the horizontal) is restricted to [−π/2,π/2] . Bounds on the remaining parameters, states, and controls are left sufficiently large so as to be effectively unconstrained. Through this implementation, the optimizer can choose any symmetrical contact sequence and the duty factor for a given speed and stride length.

Optimal gaits are found by trajectory optimization with direct collocation using GPOPS-II (v. 2.3) (Patterson and Rao, 2014) and SNOPT (v. 7.5) (Gill et al., 2005; Gill et al., 2015) in MATLAB 2020b. For each parameter combination, we keep only the best local optimum from a minimum of 50 random initial guesses. The custom software used to generate symmetrical solutions is available on GitHub (Polet, 2021a).

In the baseline unconstrained case, we compared different force-rate penalties but allowed the optimizer to choose its own track phase. In the constrained case, we forced the optimizer to use the track phase to match the fossil trackway trends. We also performed a sensitivity analysis with increasing forelimb length, leaving the track phase unconstrained. In all cases, diagonal sequence and lateral sequence gaits are equivalent, as the model is planar. The ipsilateral limb phase ( $\phi_{L}$ , the time from hindlimb contact to ipsilateral forelimb contact divided by the stride period) from optimization results for the dog model was modulated to the range $0<\phi_{L}<0.6$ , while those for the *Batrachotomus* model were modulated to $0.4\;<\phi_{L}<1$ .

### Morphology: Domestic Dogs and *Batrachotomus*

Model track phase predictions were tested against gait and footfall data readily available for Belgian Malinois dogs (Maes et al., 2008), using the body proportions specified by Polet and Bertram (2019) and the MOI specified by Polet (2021b). *Batrachotomus* dimensions and inertial properties were determined from a volumetric model (Bishop et al., 2020). This in turn was based on the skeletal reconstruction at the Staatliches Museum für Naturkunde Stuttgart. As manus and pes material is fragmentary for this species, the autopodia were partially reconstructed by the museum staff based on closely related species, and we assumed that this autopodial morphology was sufficiently accurate for our purposes. Gower and Schoch (2009) noted that specimen SMNS 90018 preserves two fragmentary metacarpals and phalanges of the right manus, and then “four metatarsals, several phalanges, and a few distally incomplete unguals” of which only the fifth metatarsal’s identity was certain. Yet what is preserved of the general autopodial morphology is consistent with the following: (1) the reconstructed, mounted museum display specimen’s morphology, having a relatively small manus vs. the pes; and (2) the general autopodial morphology of other “rauisuchian”-grade Pseudosuchia (also with smaller manus vs. pes). The model was posed in an approximate standing posture to determine leg lengths and pitch MOI about the COM (Figure 2; Table 1). The distance from the hip to COM along the glenoacetabular axis, divided by $L_{B},$ was the “COM forelimb bias,” presumed to represent percentage weight support in *Batrachotomus* (e.g., see Henderson, 2003; Willey et al., 2004). COM forelimb bias in dogs was derived from Griffin et al. (2004).

**TABLE 1:** Body parameters used as inputs to the models.

*Body Parameter*	Symbol	Batrachotomus	Belgian Malinois dog
Pes-acetabulum length	$L_{H}$	0.76 m	0.48 m
Glenoacetabular distance (GAD)	$L_{B}$	0.80 m	0.48 m
Horizontal GAD	$L_{Bx}$	0.74 m	0.48 m
Manus-glenoid length	$L_{F}$	0.46 m	0.44 m
Pes length	$L_{P}$	0.26 m	N/A
Manus length	$L_{M}$	0.15 m	N/A
COM forelimb bias	$M_{F}^{'}$	0.31	0.63
Mass	M	142 kg	28 kg
Pitch MOI about COM	I	69 kg m^2^	1.35 kg m^2^
Murphy number	$\hat{I}$	3.0	0.84

### Trackway Data

The ichnotaxa *Isochirotherium* and *Brachychirotherium* have usually been assigned to a large pseudosuchian trackmaker, possibly a “rauisuchian” such as *Batrachotomus* or a close relative (Diedrich, 2015; Apesteguía et al., 2021; Hminna et al., 2021; Klein and Lucas, 2021). Trackway data of interest include stride length, pes and manus length, and ipsilateral pes to manus distance within a footprint “set.” For each trackway of interest, we aggregated all of the above trackway data. For example, all pes lengths were used to calculate mean pes length, even if some were not associated with a manus. These data were gathered from published sources. While these sources on occasion assigned trackways to ichnospecies, for the sake of this analysis, we grouped ichnotaxa to the ichnogenus level.

#### Data from Tables

Where available, published table data were used (Petti et al., 2009; Diedrich, 2012; Diedrich, 2015). The manus–pes distance was always reported as inter-print distance (IPD). In this case, we calculated midpoint–midpoint distance as (Pes Length+Manus Length)/2 +IPD .

Petti et al. (2009) assigned four trackways to *Brachychirotherium* (identified as BsZ-A, -D, -E, and -F). Only BsZ-A and BsZ-D included manus–pes distance, and so are included in the present study. Diedrich (2012) reported three *Isochirotherium* trackways in Table 1. We interpreted the values therein as means. No variational statistics were reported, apart from number of manus/pes sets. Diedrich (2015) reported a single *Isochirotherium* trackway in their Table 1 with data for each manus/pes set. Stride lengths were reported in this table for every third manus/pes set.

#### Data from figures

Additional trackway data were extracted from published orthogonal-view photographs or traces of trackways using ImageJ (Abramoff et al., 2004). In general, for each pes print, we digitized the tip of the third digit and the caudal-most point of the fifth digit, where the craniocaudal axis was defined to lie along the axis of the third digit (Figure 1). In the cases where the fifth digit was missing, the caudal-most point of the metatarsal pad was used instead. This process was repeated for the manus tracks, except in the case of *Brachychirotherium*. Because of the rotation of the manus in this latter ichnogenus, the second digit was used for the craniocaudal axis, and the tip was digitized accordingly.

The midpoints for each manus or pes were calculated, and the stride vector was defined as the displacement between midpoints of successive pes footfalls on the same side ( $\vec{D})$ . The midpoint was chosen as it likely represents the average center of pressure for footfalls and reduces the reliance on accurately measuring single points. The manus–pes midpoint vector is the displacement from pes to manus midpoint ( $\vec{d})$ . The manus–pes distance was then calculated as the length of the projection of this vector onto the average stride vector for the trackway $\left(\vec{d}\cdot {\vec{D}}_{av}/||{\vec{D}}_{av}||\right)$ .

Clark and Corrance (2009) presented photos of single manus–pes sets of *Isochirotherium*. These were digitized as shown above. The set from their Figure 7B was associated with trackway BWF_5 data in Table 1 of the study by Clark et al. (2002), where the trackway was at first assigned to *Chirotherium*. The trackway set from Figure 6C of Clark and Corrance (2009) was associated with trackway SLID_1 data in their Table 1. However, the digitized pes in this case had a much smaller length (0.13 m) than the smallest reported value in their Table 1 (0.21 m). Based on the trackway photo (Figure 6 in Clark et al., 2002), the pes was the sixth print of SLID_1 in their Table 1, and the caudal portion of the print may have been cropped out of the photo in Figure 6C of Clark and Corrance (2009). To correct this, we extended the caudal point by the length of pes 6 reported in their Table 1 (0.28 cm). As there were no orthogonal views of either trackway available, no projection to stride could be performed, and the absolute distance from pes to manus midpoint was used instead. The pes length data for the final print in BWF_5 were excluded, as it was poorly preserved.

Apesteguía et al. (2021) assigned four trackways to *Brachychirotherium*, identified as R1, R2-t1, -t5, and -t9, and presented them in their Figures 4A, 5B. Trackway R1 includes a trailing left manus with no associated pes. Therefore, a manus–pes midpoint distance to the next left pes was calculated, and it was subtracted from the stride length to get the pes–manus midpoint distance. The same left pes was used to calculate the pes–manus midpoint distance for the next left manus print. Trackway R2-t1 appears to include a slight turn at the start of the trackway, apparent in manus–pes sets 1 and 2. The remainder of the trackway appears steady and straight. Therefore, we omitted the first two manus–pes sets for the purposes of the track phase or stride length calculations, but included them for the trackmaker size. The prints in trackway R2-t5 are relatively poorly preserved, but with enough preservation to allow identification of the ichnotaxon (*Brachychirotherium*). For this trackway, the cranial-most and caudal-most point of each print were digitized, where digits could not be discerned, with craniocaudal being defined as the trackway direction.

Klein et al. (2006) reported a single *Brachychirotherium* trackway in their Figure 7. As the digits of the first manus could not be discerned, the cranial-most portion of the print along the trackway direction was used instead.

Hminna et al. (2021) reported a single *Brachychirotherium* trackway in their Figure 4. There was only one set of well-preserved sequential ipsilateral pes footprints in this trackway. Therefore, sequential ipsilateral manus footprints were used to measure the stride length (total 4 in the trackway).

### Calculation of Gait Parameters

Hip height of the trackmaker was determined as mean track pes length times the ratio between hip height and pes length of the *Batrachotomus* model. The non-dimensional speed ( $U_{H}^{\prime }=U/\sqrt{gH}$ ) was estimated from the non-dimensional stride length ( $D_{H}^{\prime }=D/H$ , where D is the stride length) by Alexander’s (1976) dynamic similarity relation, as follows:

$$\begin{array}{c} U_{H}^{\prime }\mathrm{=0}\mathrm{.25}D_{H}^{\mathrm{\prime 1}\mathrm{.67}} \end{array}.$$ (2)

The trackway stride length and speed were inputs of the model, while the track phase was compared to optimization predictions. An analytical approximation (Stevens et al., 2016) for the track phase ( $\phi_{T}$ ) as a function of the ipsilateral limb phase ( $\phi_{L}$ ), with $L_{Bx}$ being the horizontal glenoacetabular distance, was performed as follows:

$$\begin{array}{c} \Phi_{T}\mathrm{=mod}\left(\Phi_{L}+L_{\mathrm{Bx}}/D,1\right) \end{array}.$$ (3)

This was also compared to trackway and optimization results. $L_{Bx}$ is derived from $L_{B}$ , the absolute glenoacetabular distance, as $L_{Bx}=\sqrt{L_{B}^{2}-{\left(L_{H}-L_{F}\right)}^{2}}$ in standing (Figure 2).

$L_{Bx}$ can be estimated directly from trackways if periods of quadruple stance are assumed (when all limbs are simultaneously in contact). For a trot, this parameter is given as the distance from the midpoint between left and right pes prints to the midpoint between subsequent left and right manus prints. For the present study, this is equivalent to the following:

$$\begin{array}{c} L_{\mathrm{Bx}}=d+D/2. \end{array}$$ (4)

See Supplementary Figure S1 for a geometric proof. While Stevens et al. (2016) demonstrated how these estimates can be ambiguous for small stride lengths, the stride lengths in the present study are sufficiently large such that there is no ambiguity about which prints would be in simultaneous contact (assuming quadrupedal stance).

## Results

### Belgian Malinois Dogs

**FIGURE 3:**
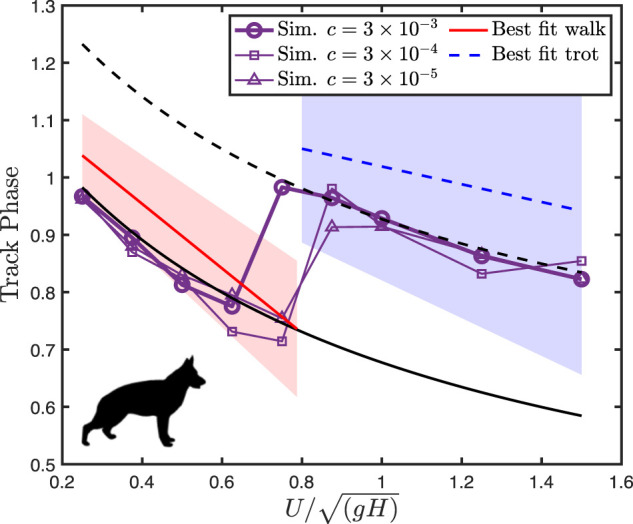
Dog track phase decreases with speed, before exhibiting a jump at the walk-trot transition. The lines of best fit to empirical data are from Maes et al. (2008), with filled areas representing two standard deviations from the mean. Black solid and dotted lines indicate $\phi_{L}=0.25$ and 0.5, respectively.

For dogs, optimization predicts a lateral sequence gait with the limb phase $\phi_{L}=0.25$ transitioning to a trot with $\phi_{L}=0.5$ , matching the natural behavior (Figure 3). The predicted track phase falls within natural variation (2 standard deviations, from Maes et al., 2008) and captures the trends of changing the track phase with speed but is consistently below the mean empirical value.

**FIGURE 4:**
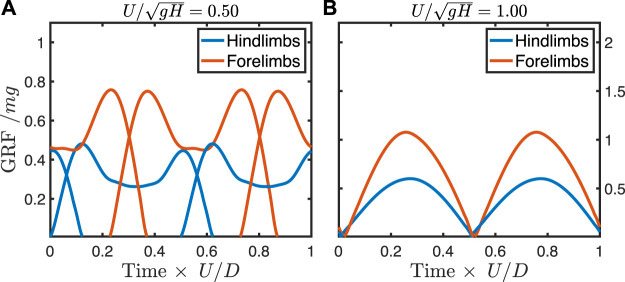
Ground reaction forces from predictive simulation at a walking speed **(A)** and trotting speed **(B)** in a dog model with force-rate penalty c′ of $3\times 10^{-3}$ . Note that any symmetrical profile is allowed, yet the simulation correctly predicts double-humped walking profiles at slow speeds and single-humped trotting profiles at faster speeds.

Varying the force-rate penalty c has little qualitative effect on the optimization results, except for the walk–trot transition point. The natural walk–trot transition point is at about $U_{H}^{\prime }=0.8.$ The transition point in simulation moves from about $U_{H}^{\prime }=0.7$ at or the highest force-rate penalty ( $c^{\prime }=3\times 10^{-3}$ ) to $U_{H}^{\prime }=0.8$ at the lowest ( $c^{\prime }=3\times 10^{-5}$ ). Overall, Polet and Bertram (2019) considered $c^{\prime }=3\times 10^{-3}$ to best match dog duty factors and ground reaction force profiles at an intermediate walking speed. The ground reaction forces at this force-rate penalty (Figure 4) qualitatively match the double-humped profile of walking (Jayes and Alexander, 1978) and the single-humped profile of trotting (Bertram et al., 2000) in dogs.

### Trackway Data

Trackways varied in size and length, with mean pes lengths varying in *Isochirotherium* from 0.22 to 0.34 m and in *Brachychirotherium* from 0.12 to 0.39 m (Table 2). *Isochirotherium* normalized speed (estimated *via* Eq. 2) varied from 0.40 to 0.79, typical for a moderate walk to fast walk or slow run. *Brachychirotherium* speeds varied more, from a slow walk at 0.31 to a fast run of 1.92. The mean manus to pes length ratio across all trackways was 0.35 ± 0.05 for *Isochirotherium* and 0.38 ± 0.09 for *Brachychirotherium* (±standard deviation).

**TABLE 2:** Summary data for *Isochirotherium* and *Brachychirotherium* trackways. Values are means ± standard deviation (sample size). Speed is normalized to presumed hip height. ET, Early Triassic; MT, Middle Triassic; LT, Late Triassic; A, Anisian; C, Carnian; N, Norian; S, “Scythian.”

Source	Track ID in source	Epoch/series, age/stage	Pes length (m)	Manus length (m)	Manus–pes distance (m)	Stride length (m)	Track phase	Estimated speed
*Isochirotherium*								
Diedrich (2012), Table 1	12	MT, A	0.34	0.12	0.24	1.68	0.14	0.60
	1	MT, A	0.22	0.10	0.17	1.28	0.13	0.79
	-	MT, A	0.24	0.08	0.19	1.30	0.15	0.70
Diedrich (2015), Table 1	-	MT, A	0.34 ± 0.02 (34)	0.12 ± 0.01 (34)	0.23 ± 0.03 (34)	1.68 ± 0.03 (11)	0.14	0.60
Clark and Corrance (2009) Figure 6C and Table 1	SLID_1	ET/MT, S/A	0.29 ± 0.03 (14)	0.1 (1)	0.28 (1)	1.2 ± 0.07 (11)	0.23	0.45
Figure 7B and Clark et al. (2002) Table1	BWF_5	ET/MT, S/A	0.28 ± 0.02 (5)	0.08 (1)	0.51 (1)	1.08 ± 0.07 (4)	0.47	0.40
*Brachychirotherium*								
Petti et al. (2009), Table 1	BsZ-A	LT, C	0.32 ± 0.02 (10)	0.09 ± 0.01 (8)	0.24 ± 0.03 (8)	1.49 ± 0.04 (8)	0.16	0.54
	BsZ-D	LT, C	0.24 ± 0 (3)	0.12 ± 0.01 (3)	0.22 ± 0.01 (3)	1.41 (1)	0.16	0.80
Apesteguía et al. (2021) Figure 1A	R1	LT	0.39 ± 0.03 (3)	0.2 (2)	0.56 (2)	1.29 (1)	0.43	0.31
Figure 5B	R2-t1	LT	0.3 ± 0.03 (7)	0.11 ± 0.02 (6)	0.26 ± 0.04 (4)	1.23 ± 0.08 (3)	0.21	0.44
	R2-t5	LT	0.24 ± 0.03 (11)	0.08 ± 0.02 (9)	0.25 ± 0.03 (9)	0.91 ± 0.04 (8)	0.27	0.39
	R2-t9	LT	0.33 ± 0.07 (5)	0.12 (2)	0.33 (2)	1.29 ± 0.04 (4)	0.26	0.41
Klein et al. (2006) Figure 7	NMMNH P-48756	LT, N	0.15 ± 0.01 (3)	0.04 ± 0.01 (3)	0.09 ± 0.01 (3)	0.98 (1)	0.09	0.96
Hminna et al. (2021) Figure 4B	CDUE 802–808	LT, C	0.12 ± 0.01 (4)	0.05 ± 0.01 (5)	0.13 ± 0.01 (4)	1.19 ± 0.04 (4)	0.11	1.92

**FIGURE 5:**
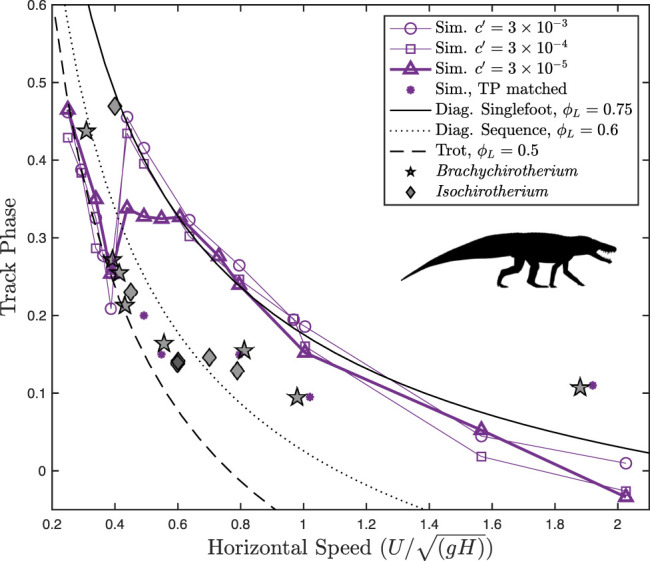
Track phase plotted against speed for *Batrachotomus* vs. fossil chirotheriid trackways. Different levels of the force-rate penalty c′ are shown as different symbols. The lowest force-rate penalty of $3\times 10^{-5}$ best fits trackway data. Both simulation and trackway data show a gradual reduction in the track phase at slow walking, corresponding to a trot based on the analytical curve (dashed line). This changes abruptly to a constant track phase with increasing speed, corresponding to the increased limb phase according to the analytical curves. Near $U_{H}^{\prime }=1$ , the theoretical upper limit for pendular walking, both simulation and trackway data exhibit a sharp reduction in the track phase, shifting toward a diagonal sequence singlefoot walk ( $\phi_{L}=0.75$ ). At intermediate speeds, the simulation consistently overestimates the trackway phase. Silhouette of *Batrachotomus* by Scott Hartman, used under a CC BY 3.0 license.

Altogether, the fossil trackways exhibit a sharp reduction of the track phase with an increasing speed for slow speeds, following the analytical line for a trot (Figure 5). Above $U_{H}^{\prime }=0.6,$ the track phase remains constant at about 0.15, until $U_{H}^{\prime }=1$ , where it falls sharply to about 0.10.

We estimated the horizontal glenoacetabular distance ( $L_{Bx}$ ) from the trackways using Eq. (4) for estimated non-dimensional speeds less than 0.4 (where quadruple limb stance may have occurred). There are only two trackways that fit this criterion, both of *Brachychirotherium* (Apesteguía et al., 2021, trackways R1 and R2-t5), which yield estimates of $L_{Bx}/L_{P}$ of 3.11 and 2.95, respectively (where $L_{P}$ is the mean pes length). The reconstructed *Batrachotomus* model has $L_{Bx}/L_{P}=2.85$ and $L_{B}/L_{P}=\;3.08$ .

Scaling the fossil trackways to the *Batrachotomus* reconstruction yields estimated $L_{Bx}$ of 0.81 and 0.79 m. The reconstruction itself gives $L_{Bx}$ = 0.74 m and $L_{B}=0.80$ m.

### *Batrachotomus* Simulation Results

Optimization predicts that *Batrachotomus* should have used a walking trot at slow speeds ( $U_{H}^{\prime }\leq 0.4)$ , transitioning to a diagonal sequence gait at faster speeds (Figure 5). The walking trot corresponds to a sharp reduction in the track phase with an increasing speed. For relatively high force-rate penalties, the transition to a diagonal sequence, singlefoot gait corresponds to a discontinuity in the track phase around $U_{H}^{\prime }=0.4$ . For the lowest force-rate penalty $(c\prime =\;3\times 10^{-5}$ ), the track phase jumps to a plateau, with the track phase remaining constant at about 0.33, while the limb phase gradually increases. Just as the limb phase reaches a diagonal sequence in singlefoot ( $\phi_{L}=0.75)$ , the track phase decreases sharply again with an increasing speed, maintaining an approximately constant limb phase.

**FIGURE 6:**
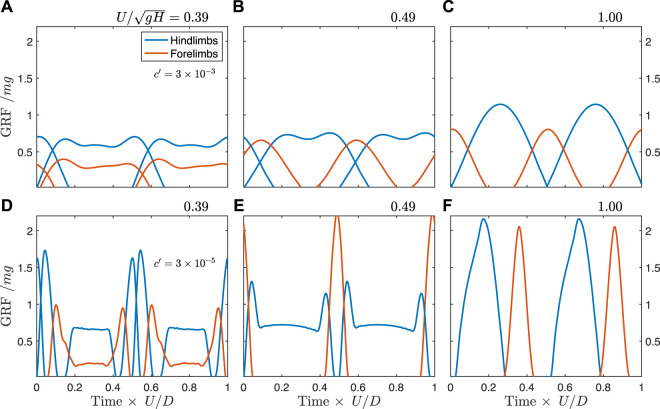
Predicted ground reaction forces from simulation for the unconstrained *Batrachotomus* model with a force-rate penalty of $3\times 10^{-3}$ (upper row) and $3\times 10^{-5}$ (lower row). **(A)** At slow speeds, a walking trot pattern is observed, with double-humped profiles typical of vaulting. **(B)** At intermediate speeds, the forelimbs transition to a single-humped, bouncing mode. **(C)** At the fastest speeds, the hindlimbs also transition to bouncing. **(D–F)** Similar behavior is also seen at a higher force-rate penalty. However, the extreme peak forces and the unloading of midstance force at some speeds **(D)** make this force-rate penalty unrealistic (see Supplementary Video S1 for animations).

At the highest force-rate penalty, the walking trot exhibits typical “vaulting” double-humped ground reaction force profiles (Figure 6A), with simultaneous contacts between fore- and hindlimbs. As speed increases, the hindlimbs continue to vault, while the single-humped force profile in the forelimbs indicates a bouncing mode (Figure 6B). At the theoretical limit for vaulting in the hindlimbs ( $U_{H}^{\prime }=1$ ), the hindlimbs also shift to bouncing (Figure 6C) (see Supplementary Video S1 for animations of these solutions). This same general shift in gait is also seen at the lowest force-rate penalty (Figures 6D–F), though the peak forces approach impulsivity, as is expected from work-minimizing optimization (Srinivasan, 2010). At walking speeds, the solutions can exhibit periods during stance where the hindlimbs are completely offloaded (Figure 6D).

**FIGURE 7:**
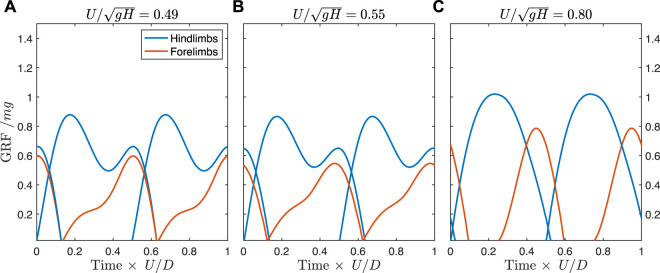
Predicted ground reaction forces for the *Batrachotomus* model constrained to match the empirical track phase. **(A)** By a speed of 0.49, the optimal solution is to use skewed ground reaction forces in the forelimbs, with semi-vaulting forces in the hindlimbs. **(B)** This pattern is preserved at the transition point where the track phase remains constant with increasing speed. **(C)** By a speed of 0.80, the solution shifts to a running tölt, similar to the unconstrained case but differing in the timing of forelimb contact. The same solution is found at faster speeds in the constrained case, but with lower duty factor and higher peak forces (see Supplementary Video S2 for animations).

When solutions are constrained to match the track phase trends of the fossil trackways, the ground reaction forces differ somewhat from the unconstrained case. Prior to the transition to a constant track phase, the optimal solution transitions away from a vaulting, walking trot, and exhibits vaulting hindlimbs with skewed ground reaction forces in the forelimbs indicating an asymmetrical (generative) bouncing mode (Figure 7A, see also Supplementary Video S2). This same gait is used at the transition to the constant track phase (Figure 7B), but gradually shifts to bouncing in both fore- and hindlimbs, similar to the unconstrained case (Figure 7C). As speed increases further, the solution remains the same, but with a lower duty factor and higher peak forces.

The constrained solutions exhibit higher costs than the unconstrained solutions (Supplementary Figure S2A). The difference in cost is largest at intermediate speeds and smaller at faster speeds. At $U_{H}^{\prime }=0.55$ , the cost of transport of the constrained case is 1.2 times that of the constrained case (Supplementary Figure S2B). At $U_{H}^{\prime }=1.92$ , this ratio diminishes to 1.01.

## Discussion

The simulation method predicts the canine track phase within natural variation, though predicted values are consistently below the mean empirical values (Figure 3). The model correctly predicts the symmetrical walking and running gaits used by dogs, the shape of their ground reaction forces (Figure 4), and the gait transition speed. This gives us some confidence in applying the model to forms where only the stride length, size, and shape can be estimated, and gait is unknown.

*Brachychirotherium* trackways follow a consistent pattern with the track phase and speed: a sharp reduction with increasing speeds below $U_{H}^{\prime }=0.6$ , followed by a constant track phase up to $U_{H}^{\prime }=1$ , at which point the track phase suddenly drops (Figure 5). *Isochirotherium* trackways also follow this pattern, except data are missing above dimensionless speeds of 0.8. This broad similarity in the track phase pattern with estimated speed justifies combining these ichnogenera for the analysis.

At all force-rate penalties, the simulation data correctly predict a walking trot at slow speeds, closely following the analytical curve. In all cases, the optimal gait transitions at around $U_{H}^{\prime }=0.4$ and shifts to a higher track phase. Not only does this indicate an increase in the limb phase from a trot to a diagonal sequence gait but it also represents a fundamental shift in the gait type. While the ground reaction force profiles of the slow-speed gait exhibit the double-humped shape characteristic of vaulting (Figures 6A,D), the faster gait is a hybrid, with vaulting hindlimbs and bouncing forelimbs (Figures 6B,E). This most closely matches the slow tölt of Icelandic horses (Biknevicius et al., 2004). Like Icelandic horses, the simulation switches to a “running tölt” at faster speeds: a four-beat symmetrical gait with single-humped ground reaction force profiles in all limbs (Figure 6C). The tölt is a rare gait in mammals (Vincelette, 2021), and unheard of in archosaurs, though it has been detected in fossil horse trackways (Renders, 1984; Vincelette, 2021).

At speeds above 0.4, however, the simulations differ markedly from fossil trackways in the track phase. Still, the trackways exhibit stark changes in the track phase, at around $U_{H}^{\prime }=0.6$ and 1.0, at similar times to when the simulations exhibit changes in gait. If the simulation is constrained to match the track phase of the fossil trackways, the solution remains qualitatively similar to the unconstrained case, inasmuch as exhibiting bouncing in the forelimbs, with vaulting in the hindlimbs at $U_{H}^{\prime }=0.6$ (Figure 7)—similar to a slow tölt—before transitioning to a bouncing mode in both limbs at $U_{H}^{\prime }=1$ — similar to a fast tölt. The stark changes in the fossil track phase at $U_{H}^{\prime }=0.6$ and 1.0, its correspondence with a change in the limb phase according to analytical relations, combined with multiple simulations predicting gait transitions near these speeds, suggest that these fossil data demonstrate a gait transition in the pseudosuchian trackmakers.

Although the fossil trackways exhibit manus placed cranially to the ipsilateral pes in a couplet, qualitatively matching modern crocodylians in a walking trot (Kubo, 2008), the long trackway stride lengths imply gaits more similar to a dissociated trot or diagonal sequence. Modern crocodylians, the closest living relatives of *Batrachotomus*, do not normally transition to a four-beat gait at faster speeds, instead either continuing to trot or transitioning to asymmetrical gaits (Hutchinson et al., 2019). Although extant crocodylians do exhibit an increase in the limb phase as the speed increases for symmetrical gaits (Supplementary Figure S3), the change is much more gradual than observed here. Crocodylians do not always walk with the lateral sequence diagonal-couplet gait (Supplementary Figure S3; Hutchinson et al., 2019) held to be ancestral for quadrupedal gnathostomes (Wimberly et al., 2021). However, these results represent the first evidence that extinct pseudosuchians exhibited different gaits than their modern relatives, and the first evidence of a gait transition in an extinct pseudosuchian.

The use of a two-beat gait at slow speeds, and a four-beat gait at fast speeds, is consistent with an analysis by Polet (2021b). The large pitch moment of inertia of *Batrachotomus* relative to its glenoacetabular distance gives it a Murphy number of 3. Above a Murphy number of 1, a four-beat run is optimal because the energetic cost of pitching the body is lower than the energetic cost of moving the body up and down, and so it is not economical to reject pitching by using a trotting gait. However, in order to maintain vaulting in a typical four-beat walk, the body must pitch, which is energetically expensive for an animal with a large pitch moment of inertia. For this reason, a walking trot is favored at slow speeds.

**FIGURE 8:**
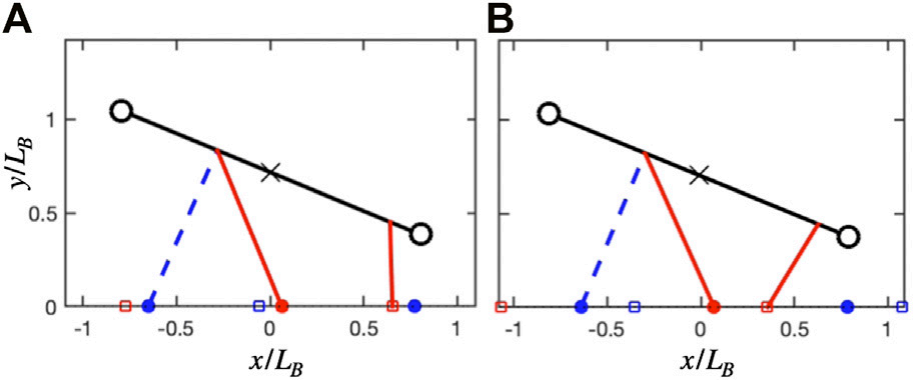
Geometry at hindlimb step transition for the unconstrained and constrained track phase cases, both at $U_{H}^{\prime }=0.49$ and $c^{\prime }=0.003.$ **(A)** In the unconstrained case, the forelimb is nearly vertical, allowing the hindlimbs to be effectively offloaded. The resulting ground reaction forces (GRF) are symmetrical, as shown in Figure 6B. **(B)** In the constrained track phase case, the shoulder is extended, so will provide a forward as well as vertical propulsive force. It can assist the hindlimbs pushoff in this position. GRF forces become asymmetrical, as shown in Figure 7A.

Both the unconstrained and constrained cases exhibit similar solutions in ground reaction forces, but these are skewed in the latter case. In the unconstrained case, the forelimb is nearly vertical when the hindlimbs are in double support (Figure 8A). By having peak force at midstance, it can effectively offload the weight of the body during the costly step transition for the hindlimbs (Schroeder and Bertram, 2018). At the same time, the symmetrical, single-hump profile acts as a distributed pseudo-elastic “collision” (Figure 6B), which minimizes the cost of limb work while managing the force-rate penalty (Ruina et al., 2005; Rebula and Kuo, 2015).

In the constrained case, the shoulder is flexed during double support (Figure 8A), meaning that any pushing forces from the leg will generate both vertical and forward propulsion of the center of mass. This is less effective for offloading weight during transfer of support, but does contribute to the pre-transfer pushoff of the hindlimbs, which mitigates work-related energetic losses (Ruina et al., 2005; Rebula and Kuo, 2015; Schroeder and Bertram, 2018). Therefore, the force in the forelimbs can increase, while the second peak in the hindlimbs can decrease (Figure 7A). The asymmetry however results in a generative (plastic) distributed “collision,” which typically requires more work than a symmetrical (pseudo-elastic) collision (Ruina et al., 2005).

The transition of trackways away from the analytical walking trot line occurs at around $U_{H}^{\prime }=0.6$ (Figure 5), well below the maximum speed for a pendular walk of $U_{H}^{\prime }=1$ (Usherwood, 2005), but matching the transition to a four-beat gait predicted by Polet (2021b) for a Murphy number of 3 in a perfectly symmetrical model (center of mass at midpoint of glenoacetabular distance, with all legs equal in length to glenoacetabular distance).

When the gait transition seems to occur in the trackway data, the ipsilateral manus and pes prints are nearly overstepping. Increasing the speed further without changing gait would result in overstepping, and would result in a collision of the ipsilateral manus and pes, unless the animal changed its limb orientations. Is it possible that the gait transition we infer here was forced due to this physical constraint, rather than any energetic consideration?

In modern crocodylians, foot collision appears to be avoided in the walking trot by changing yaw of the body so that the craniocaudal axis is not exactly aligned with the direction of motion. This results in manus prints being placed slightly to the left or right of pes prints (Kubo, 2008; see Figures 2A,E therein). This strategy is also employed by dogs when trotting, as can be seen in traces (Murie and Elbroch, 2005). There is no *a priori* reason to expect that “rauisuchians” such as *Batrachotomus* would have been unable to yaw their bodies in the same way in order to continue to employ a walking trot at higher speeds. Likewise, the planar model we use here is not constrained to avoid collisions of ipsilateral legs or feet. The transition, in this case, is driven completely by energetic considerations.

The earlier transition from a trot in the simulations (around $U_{H}^{\prime }=\;0.40$ ) seems to be driven by the relatively short forelimbs of *Batrachotomus* (61% of hindlimb length). A 10% increase in the model’s forelimb length increases the transition speed from 0.40 to 0.45, while a 50% increase changes it to 0.49 (Supplementary Figures S4, 5), simultaneously decreasing the track phase value at the transition speed. This may reflect a difference between the morphology of the real trackmakers and *Batrachotomus*. The reconstruction has a manus to pes length ratio of 0.58, compared to 0.35–0.38 in the trackways. This is also consistent with fossil footprint morphology, with the manus prints appearing digitigrade or semi-digitigrade in forelimbs (Diedrich, 2015; Klein and Lucas, 2021), roughly matching modern crocodylians (Hutson and Hutson, 2015), while *Batrachotomus* is interpreted as plantigrade. It is also possible that *Batrachotomus* in life exhibited a less flexed elbow than interpreted here, thereby increasing the effective forelimb lengths (Figure 2).

This is perhaps also reflected in the ratio between estimated horizontal glenoacetabular distance and pes length ( $L_{Bx}/L_{P}$ ) in *Brachychirotherium* trackways (2.95–3.11), which match the *Batrachotomus* reconstruction more closely when absolute glenoacetabular distance is used ( $L_{B}/L_{P}=$ 3.08) rather than $L_{Bx}/L_{P}$ (2.85). This may result from a more equal height of glenoid and acetabulum than previously assumed, but it could equally be due to morphological differences between *Batrachotomus* and *Brachychirotherium*, or uncertainties in the pedal reconstruction of *Batrachotomus* used in this study.

While the simulations provide evidence of a gait transition in the trackmakers, they do not predict the track phase well except at slow speeds. There may be several reasons for this discrepancy. First, the model may not accurately capture the energetics of gait alternatives in “rauisuchians.” This may be due to neglecting important morphological features (e.g., legs with inertia), or physiological characteristics (e.g., muscles with force–velocity characteristics). Second, the trackmakers may not have followed the simple stride length to speed relationship proposed by Alexander (1976; based mainly on mammals). One reason may be due to a relatively reduced hip flexion and extension in some “rauisuchians” (Nesbitt et al., 2013), which was not considered in the present analysis. Third, the trackmaker may not resemble *Batrachotomus* in proportion, mass distribution, or other key areas. Finally, it is possible that energetics were not key determinants of locomotion for these trackmakers. The soft substrate where these tracks were formed, for example, could affect gait choice and phase relationships. Future developments in predictive simulation of pseudosuchian locomotion can address some of these issues by adding realism and evaluating the models within extant crocodylians.

## Conclusion

We applied a planar, generalized quadrupedal model to the gait of *Batrachotomus kupferzellensis*, an extinct crocodile-line (pseudosuchian) archosaur. We compared our predictions to fossil trackways putatively left by close relatives of *Batrachotomus*. When optimized to minimize leg work and force rate squared, the model correctly predicted a sharp reduction in the track phase with speed, corresponding to a trot, at low speeds. Next, the model predicted a transition to a four-beat walk similar to a slow tölt, near the region where the fossil trackways deviated from the walking trot trajectory and appeared to transition to a diagonal sequence gait. Finally, when the fossil trackways exhibited another sharp transition in the track phase, the model predicted a transition to a four-beat gait similar to a fast tölt. This represents the first evidence of a gait transition in an extinct pseudosuchian, and the first evidence that “rauisuchians” like *Batrachotomus* may have exhibited some gaits different from modern crocodylians.

Because *Batrachotomus* is inferred to have had features of both modern crocodylians and mammals, trajectory optimization provides an opportunity to understand their gait where no direct analogue exists. According to the optimization results, the large pitch moment of inertia and erect limb posture of *Batrachotomus* made a tölt-like gait favorable, something not seen in any archosaurian group today and rare in mammals. This raises exciting questions, such as when this suite of gaits evolved in pseudosuchians or how often it did, and what the ancestral state was for Archosauria (birds, crocodiles, and all extinct descendants of their common ancestor, including Mesozoic dinosaurs). More sophisticated three-dimensional models incorporating lateral motions and more realistic morphology (e.g., Bishop et al., 2021) or analysis of neuromuscular control and stability may provide further insight.

## Data Availability

The raw data supporting the conclusion of this article will be made available by the authors, without undue reservation.
