# Supplementary material for: Estimating Gaits of an Ancient Crocodile-Line Archosaur Through Trajectory Optimization, With Comparison to Fossil Trackways
Source: Front Bioeng Biotechnol. 2022 Feb 3;9:800311. doi: 10.3389/fbioe.2021.800311 (PMC8852800; doi:10.3389/fbioe.2021.800311)
Supplement: Supplementary file 1 [file Presentation1.pdf]

Supplementary Material

1 Supplementary Data

All raw trackway data are included in Supplementary_Data_1.xlsx. Note that the “source” column contains only first author and year for the associated paper. Refer to Table 1 in the main manuscript for more detailed references.

Data for *Chirotherium* from Avanzini and Cavin (2009, their figure 3) are included. The third digit of the second and third pes print was not well preserved, and so the tip of the second digit was used instead. This trackways was originally assigned to *Isochirotherium* and later reassigned to *Chirotherium* (Klein et al., 2016).

2 Supplementary Video Captions

Supplementary Video S1 Animations of the unconstrained *Batrachotomus* model solutions at various nondimensional speeds. Animations correspond to ground reaction forces shown in Figure 6A, B and C. First, animations are shown at “real” time, based on the relationship of Alexander (1976), then in slow motion. Note uncertainty in stride frequency is high, especially at low stride lengths, and the frequencies shown here are likely higher than they would be in life. Leg opacity is scaled to the peak force experienced by any one leg. “X” shows center of mass (COM) location, and circles show radii of gyration from the COM.

Supplementary Video S2 Animations of the trackphase-constrained *Batrachotomus* model solutions at various nondimensional speeds. Animations correspond to ground reaction forces shown in Figure 7. First, animations are shown at “real” time, based on the relationship of Alexander (1976), then in slow motion. Note uncertainty in stride frequency is high, especially at low stride lengths, and the frequencies shown here are likely higher than they would be in life. Leg opacity is scaled to the peak force experienced by any one leg. “X” shows center of mass (COM) location, and circles show radii of gyration from the COM.

3 Supplementary Figures

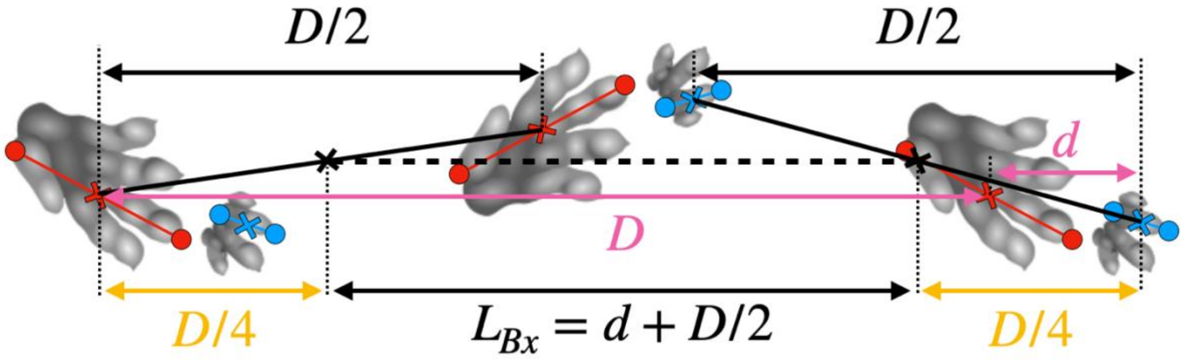

Supplementary Figure S1 Schematic showing how horizontal glenoacetabular distance ($\boldsymbol{L_{Bx}}$) is estimated. $\boldsymbol{L_{Bx}}$ (dotted line) is the distance between the midpoint of left and right pedes and subsequence left and right manus. This is equal to the sum of the pink lengths minus yellow lengths. Note that, in a perfectly symmetrical gait, sequential left and right pedes (or manus) are exactly half a stride apart ($\boldsymbol{D/2}$). While single strides will not be perfectly symmetrical, we expect trackways of steady locomotion to tend towards symmetry with increasing trackway length.

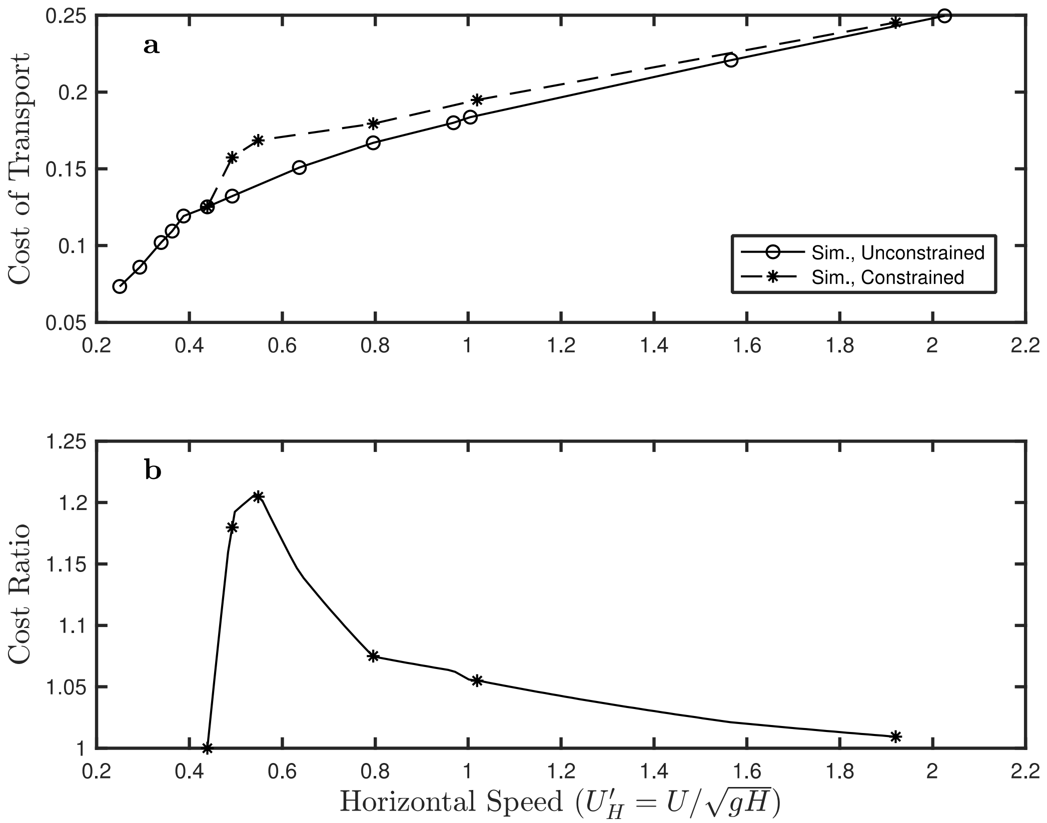

Supplementary Figure S2 Cost of transport compared between unconstrained solutions (circles) and solutions constrained to match the track phase of the fossils (stars). (a) The constrained solutions are consistently more costly than unconstrained, but the difference diminishes with higher speeds. (b) The ratio between the cost of constrained and unconstrained simulations peaks at about 1.2, before decaying to less than 1.01.

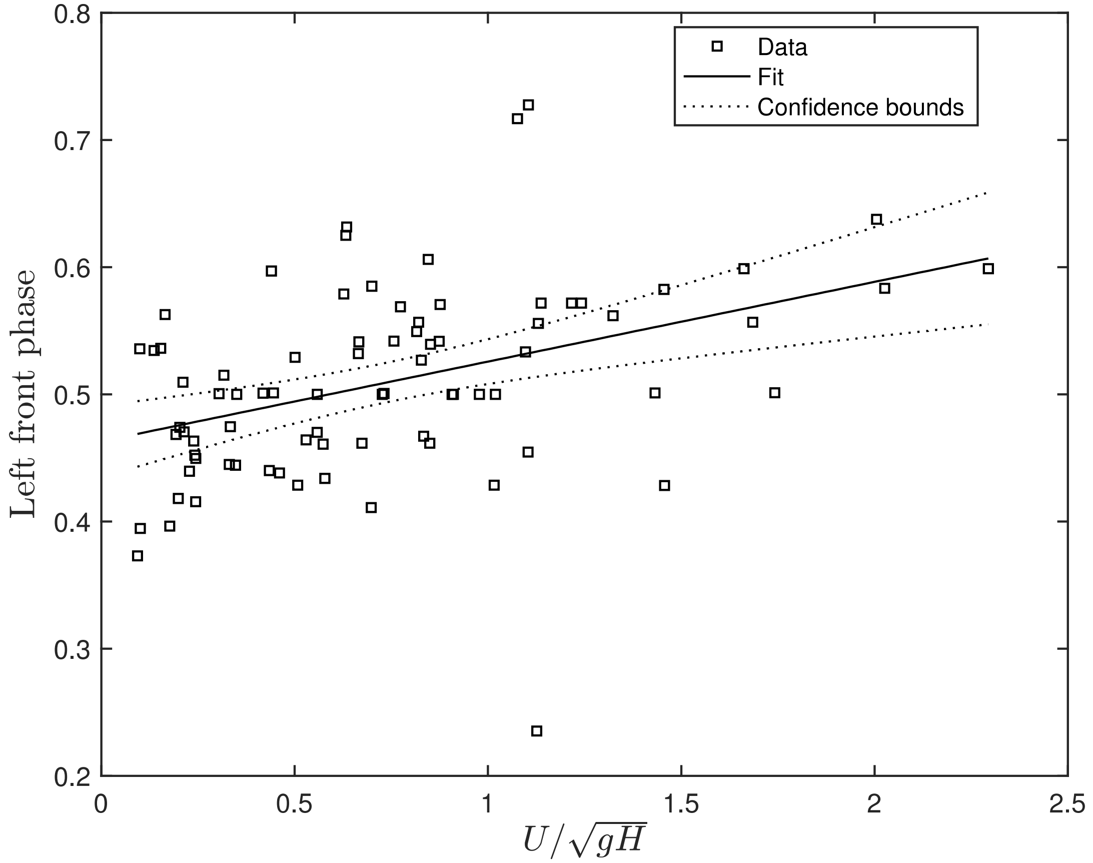

Supplementary Figure S3 Phase of the left forelimb contact relative to left hindlimb contact, for symmetrical gaits of crocodylians. Symmetrical gaits were derived from data published by Hutchinson et al. (2019), and available at https://doi.org/10.1038/s41598-019-55768-6. Symmetry was defined as the phase of the right forelimb relative to the left forelimb being between 0.4 to 0.6 (and the same criterion for the hindlimbs). The phase of the left front limb ($\boldsymbol{\phi_{L,LF}}$) increases linearly with speed (least squares best fit, $\boldsymbol{\phi_{L,LF} = 0.06U'_H + 0.46}$, $\boldsymbol{p < 0.001}$, $\boldsymbol{N = 80}$), but the variation in the data is not well explained by a linear model ($\boldsymbol{R^2 = 0.07}$).

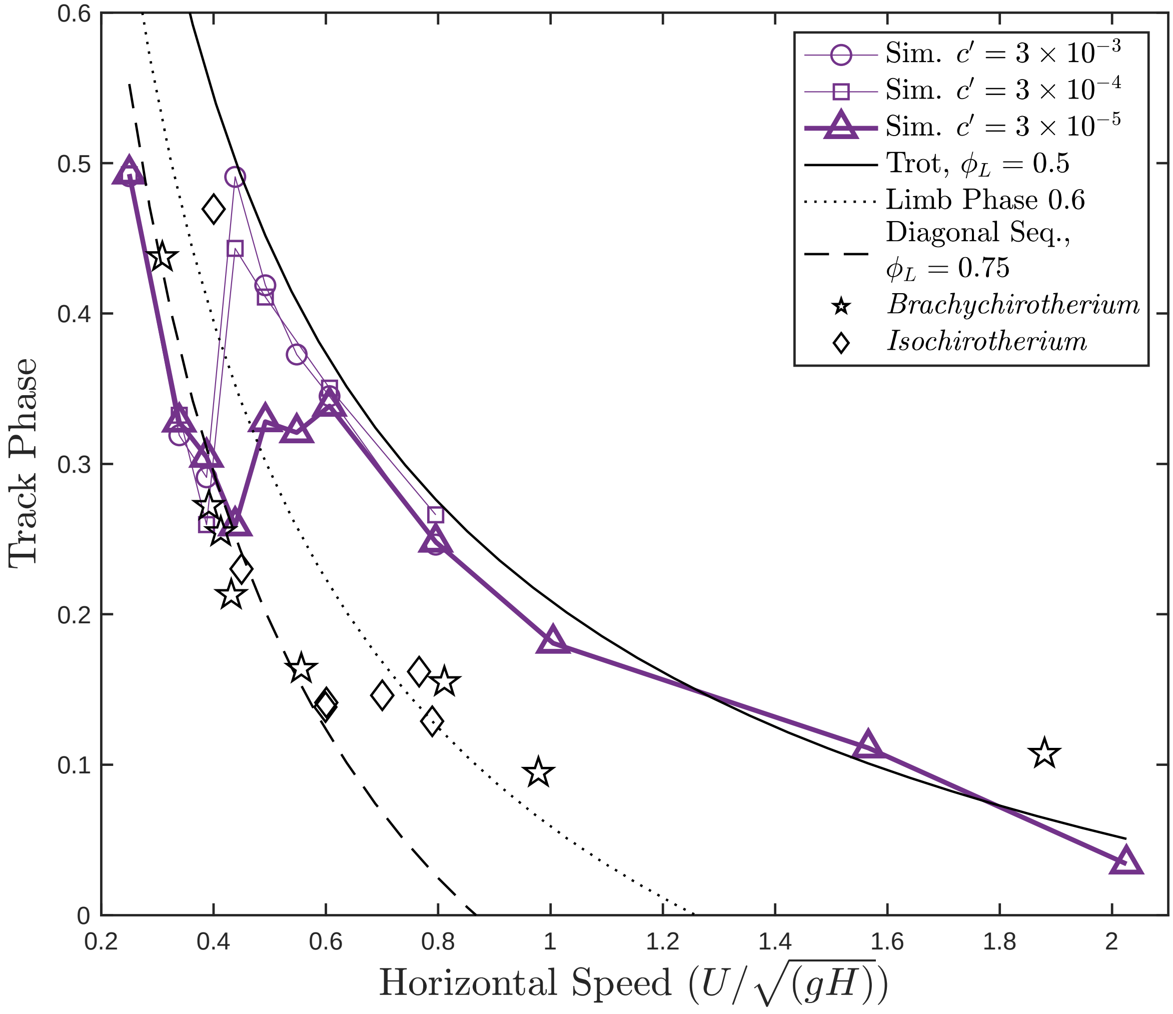

Supplementary Figure S4 Track phase vs speed for simulations with 10% increase in forelimb length.

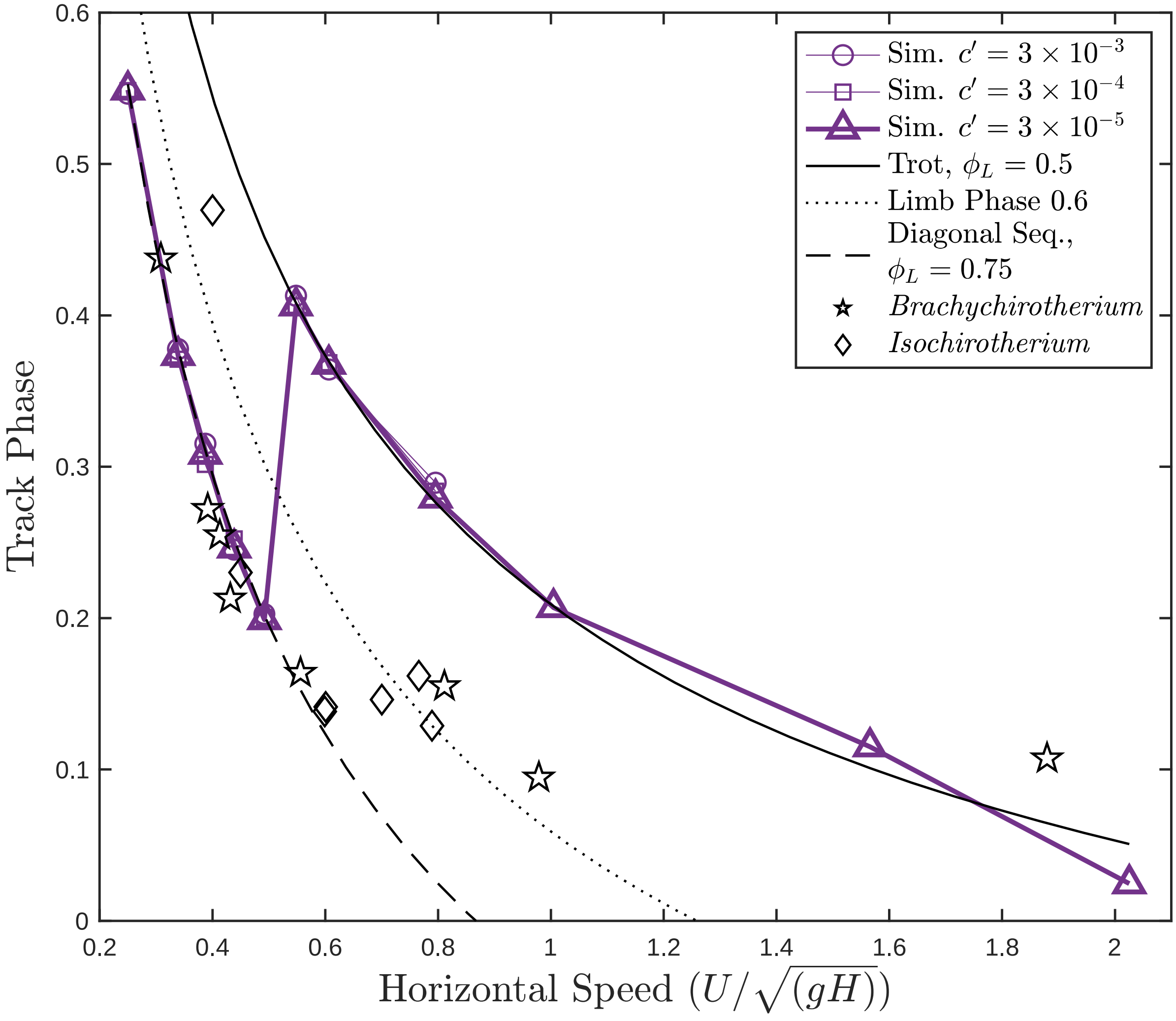

Supplementary Figure S5 Track phase vs speed for simulations with 50% increase in forelimb length.

References

Avanzini, M., and Cavin, L. (2009). A new *Isochirotherium* trackway from the Triassic of Vieux Emosson, SW Switzerland: stratigraphic implications. *Swiss J. Geosci.* 102, 353–361. doi:10.1007/s00015-009-1322-4.

Klein, H., Wizevich, M. C., Thüring, B., Marty, D., Thüring, S., Falkingham, P., et al. (2016). Triassic chirotheriid footprints from the Swiss Alps: ichnotaxonomy and depositional environment (Cantons Wallis & Glarus). *Swiss J. Palaeontol.* 135, 295–314. doi:10.1007/s13358-016-0119-0.
